# Systematic review and meta-analysis of photon radiotherapy versus proton beam therapy for pediatric intracranial ependymoma: TRP-ependymoma 2024

**DOI:** 10.1016/j.heliyon.2024.e40372

**Published:** 2024-11-14

**Authors:** Masashi Mizumoto, Sho Hosaka, Kei Nakai, Yinuo Li, Yoshiko Oshiro, Takashi Iizumi, Takashi Saito, Masako Inaba, Hiroko Fukushima, Ryoko Suzuki, Shosei Shimizu, Kazushi Maruo, Hideyuki Sakurai

**Affiliations:** a Department of Radiation Oncology, University of Tsukuba, Tsukuba, Ibaraki, 305-8576, Japan; b Department of Pediatrics, University of Tsukuba Hospital, Tsukuba, Ibaraki, 305-8576, Japan; c Department of Radiation Oncology, Tsukuba Medical Center Hospital, Tsukuba, Ibaraki, 305-8558, Japan; d Department of Child Health, Institute of Medicine, University of Tsukuba, Tsukuba, Ibaraki, 305-8576, Japan; e Department of Pediatric Radiation Therapy Center / Pediatric Proton Beam Therapy Center, Hebei Yizhou Cancer Hospital, 072750, China; f Department of Biostatistics, Institute of Medicine, University of Tsukuba, Tsukuba, Ibaraki, 305-8576, Japan

**Keywords:** Ependymoma, Brain, Proton, Radiotherapy, Meta-analysis, Systematic review, TRP

## Abstract

**Introduction:**

Proton beam therapy (PBT) may reduce the number of adverse events in treatment of patients with pediatric cancer. However, it is difficult to evaluate whether the actual therapeutic effect is truly equivalent to that of photon radiotherapy. To compare photon radiotherapy and PBT, a meta-analysis and systematic review were performed.

**Methods:**

The meta-analysis used papers from 1990 to 2023 in which postoperative local photon radiotherapy or PBT was performed for pediatric intracranial ependymomas. Fifteen articles (5 PBT, 9 photon radiotherapy, one both) were selected based on administration of radiotherapy as local irradiation.

**Results:**

Among the 15 chosen articles, the 1- to 5-year overall survival (OS) rates (photon radiotherapy vs. PBT) were 95.4 % (95 % confidence interval (CI) 92.8–97.1 %) vs. 97.2 % (95.7–98.2 %); 88.3 % (85.0–90.9 %) vs. 93.5 % (91.4–95.1 %); 81.2 % (76.9–84.8 %) vs. 91.1 % (88.4–93.2 %); 76.9 % (71.2–81.6 %) vs. 86.1 % (81.9–89.4 %); and 73.8 % (68.3–78.5 %) vs. 84.7 % (79.9–88.5 %), respectively. The 1- to 5-year local control (LC) rates (photon radiotherapy vs. PBT) were 90.9 % (95 % CI 83.9–94.9 %) vs. 91.0 % (88.7–92.9 %); 81.5 % (68.9–89.4 %) vs. 85.7 % (82.0–88.6 %); 77.3 % (62.8–86.8 %) vs. 82.6 % (79.1–85.5 %); 74.6 % (57.7–85.6 %) vs. 78.3 % (71.6–83.5 %); and 72.6 % (51.4–85.8 %) vs. 79.0 % (73.4–83.5 %), respectively. The meta-regression analysis identified relationships of modality (photon radiotherapy vs. PBT), age at irradiation, pathology (Grade 2 vs. Grade 3), and tumor removal (complete resection vs. none) with significantly better 3-year OS after PBT and better 1- to 5-year LC at a younger age.

**Conclusion:**

In postoperative local irradiation of ependymomas in children, proton beam therapy had outcomes comparable to those of photon radiotherapy.

## Highlights

• To our knowledge, this is the first meta-analysis comparing postoperative photon radiotherapy and proton beam therapy (PBT) for pediatrics intracranial ependymomas.
• 3 and 5-year overall survival rates (photon radiotherapy vs. PBT) of 81.2 % (76.9–84.8 %) vs. 91.1 % (88.4–93.2 %); and 73.8 % (68.3–78.5 %) vs. 84.7 % (79.9–88.5 %), respectively. And 3 and 5-year local control rates (photon radiotherapy vs. PBT) were 77.3 % (62.8–86.8 %) vs. 82.6 % (79.1–85.5 %); and 72.6 % (51.4–85.8 %) vs. 79.0 % (73.4–83.5 %), respectively.
• In postoperative local irradiation of ependymomas in children, proton beam therapy had outcomes comparable to those of photon radiotherapy.

## 1 Introduction

Ependymomas are brain tumors that predominantly occur at less than 15 years of age and are located below the infratentorial region (two-thirds of cases) and in the supratentorial region (one-third of cases) [1,2]. Resection surgery is normally performed, with the need for postoperative treatment determined by pathological findings and extent of resection [[1], [2], [3]]. Radiation therapy (RT) is commonly used postoperatively in cases with grade 2 pathology findings and incomplete resection, or in cases with grade 3 pathology [4,5]. Ependymomas with spinal cord metastases have also been treated with craniospinal irradiation [6,7]. Local irradiation is now often chosen after surgery in the absence of obvious disseminated lesions, and Merchant et al. have shown good outcomes after local irradiation, with 5-year rates of overall survival (OS) and local control (LC) of about 80 % each [8]. Chemotherapy may be used in the presence of clear residual disease or to delay the start of irradiation, but there is no clear indication for chemotherapy [4,5,8].

Proton beam therapy (PBT) has better dose concentration than conventional photon RT, and good results have been reported for hepatocellular carcinoma and other cancers [9,10]. In particular, PBT minimizes the risk of secondary cancers and growth retardation due to low dose exposure compared to photon RT [11]. However, because pediatric tumors are rare, it is difficult to determine if PBT gives similar outcomes to those with photon RT. To obtain more accurate results from limited evidence, the Tsukuba Review Project (TRP) was launched by the Department of Radiation Oncology and Department of Biostatistics. In this study, a meta-analysis was performed to compare photon RT with PBT for ependymomas. The purpose of this study was to confirm that PBT is not inferior to photon RT, assuming that PBT is administered under the same treatment policy as photon RT.

## 2 Methods

### 2.1 Selection criteria for meta-analysis

The review complied with the Preferred Reporting Item for Systematic Reviews and Meta-Analysis (PRISMA) guidelines and recommendations [12]. Only English language articles were included. All articles were screened by two reviewers. The inclusion criteria were [1]: clinically diagnosed intracranial ependymoma [2], treatment with radical RT (photon RT or PBT) [3], RT mainly used as local treatment [4], OS, progression free survival (PFS) or LC rate with RT can be confirmed in the manuscript, and [5] ≥30 treatment results are specified.

**Fig. 1:**
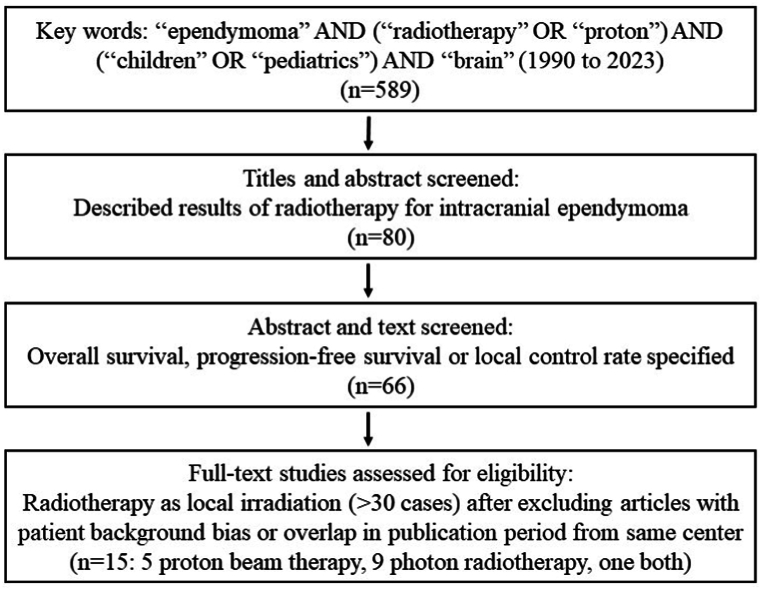
Manuscript selection process.

A PubMed search for “ependymoma” AND (“radiotherapy” OR “proton”) AND (“children” OR “pediatrics”) AND “brain” from 1990 to 2023 identified 589 articles. Of these, 80 describing treatment results of RT for intracranial ependymoma were selected based on the title or abstract, and 66 of these articles were found to report OS, PFS or LC rate in the abstract or text. Finally, 15 articles (5 PBT, 9 photon RT, one both) were selected based on administration of RT as local irradiation, after exclusion of articles with significant patient background bias and publications from the same center with overlapping periods. These articles [8,13,14,15,16,17,18,19,20,21,22,23,24,25,26] are shown in Table 1. The manuscript selection process is summarized in Fig. 1. Data were collected from each manuscript for authors, publication year, country, study design, number of patients, deaths, all recurrences, local recurrences, follow-up period, 1- to 5-year OS rates, 1- to 5-year LC rates, age, gender, gross total resection rate, pathology grade (2 vs. 3), chemotherapy rate, total dose, and treatment modality (photon RT vs. PBT). If the text did not indicate the 1- to 5-year OS and LC rates, these were estimated from figures. Within the limits specified in the manuscript, the irradiation method of photon RT was either Three-Dimensional Conformal Radiation Therapy (3D-CRT) or Intensity Modulated Radiation Therapy (IMRT).

**Table 1:** List of selected manuscripts.

Author	Year	Modality	Study design	n	GTR rate	G3 rate	Infra rate	3 y OS (%)	5 y OS (%)
Peters	2022	Proton	R	105	71.4	75.2	81.9	91	79
Indelicato	2021	Proton	R	386	85.0	64.5	71.0	91	85
Indelicato	2018	Proton	R	179	84.9	67.0	66.5	90	–
Sato	2017	Proton	R	41	92.7	80.5	75.6	97	88
Ares	2016	Proton	R	50	66.0	92.0	72.0	88	84
Macdonald	2013	Proton	R	70	65.7	47.1	72.9	95	–
Liu	2022	Photon	R	30	–	–	–	62	49
Ritzmann	2022	Photon	R	74	44.6	47.3	63.5	77	69
Napieralska	2021	Photon	P	74	59.5	48.6	63.5	92	83
Ducassou	2018	Photon	R	202	85.6	63.9	72.8	77	71
Sato	2017	Photon	R	38	76.3	81.6	60.5	81	68
Massimino	2016	Photon	P	158	69.0	52.5	69.0	86	81
Kim	2013	Photon	R	72	58.3	43.1	77.8	86	77
Garvin	2012	Photon	P	84	51.2	21.4	58.3	79	71
Merchant	2009	Photon	P	153	81.7	55.6	79.7	86	83
Shu	2007	Photon	R	47	61.7	31.9	63.8	79	66

P: prospective study, R: retrospective study, GTR: gross total resection (%), G3: pathological Grade 3 (%).

### 2.2 Statistical analysis

Random effects meta-analyses of 1- to 5-year OS and LC rates were performed for each modality, and forest plots were drawn. Missing values for accuracy were imputed using the number of cases, risk set size at each year, and mean dropout rate. Heterogeneity was evaluated by I-square statistics in each meta-analysis. Random-effects meta-regression with modality as an explanatory variable were performed for each outcome to compare among modalities. The analyses were performed using R (R Core Team, Vienna, Austria) and its meta package [27].

## 3 Results

**Fig. 2:**
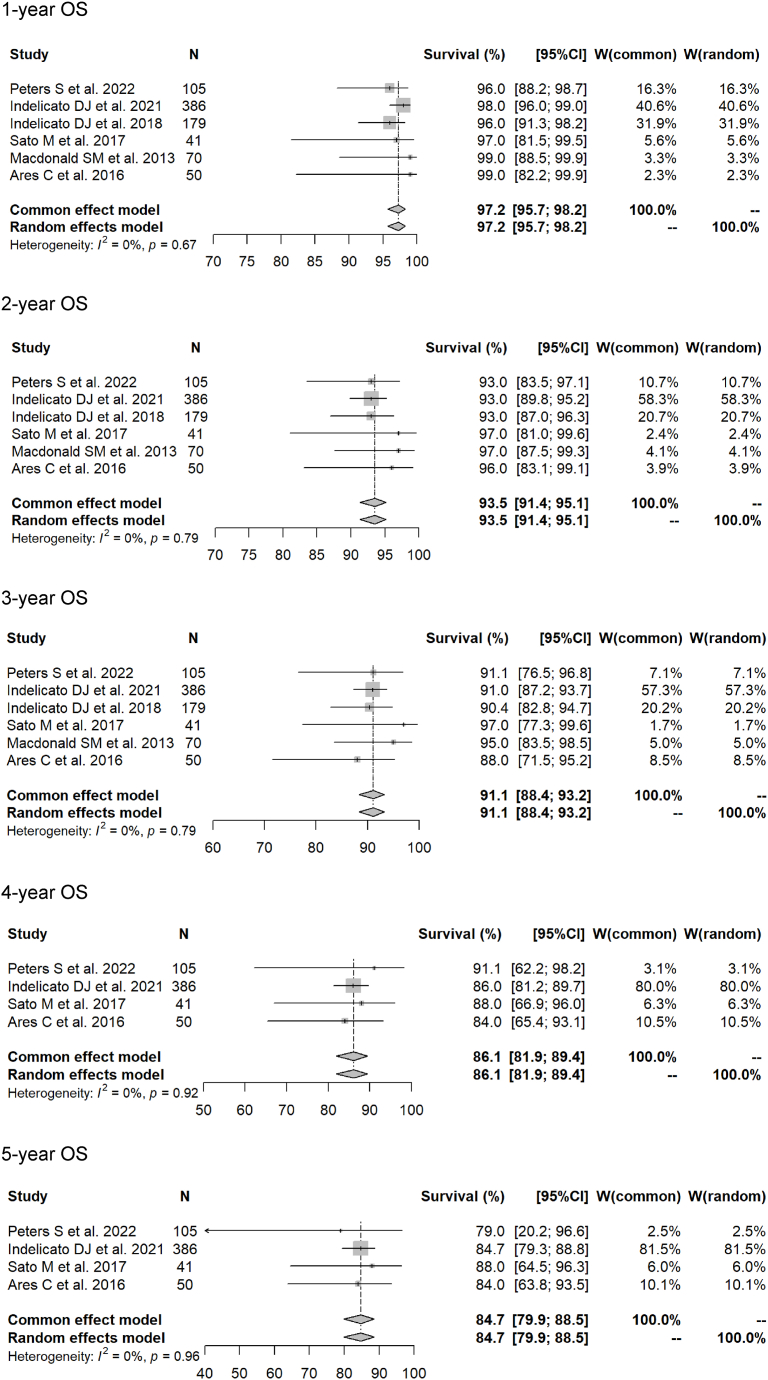
Forest plots of 1- to 5-year overall survival rate for proton beam therapy.

**Fig. 3:**
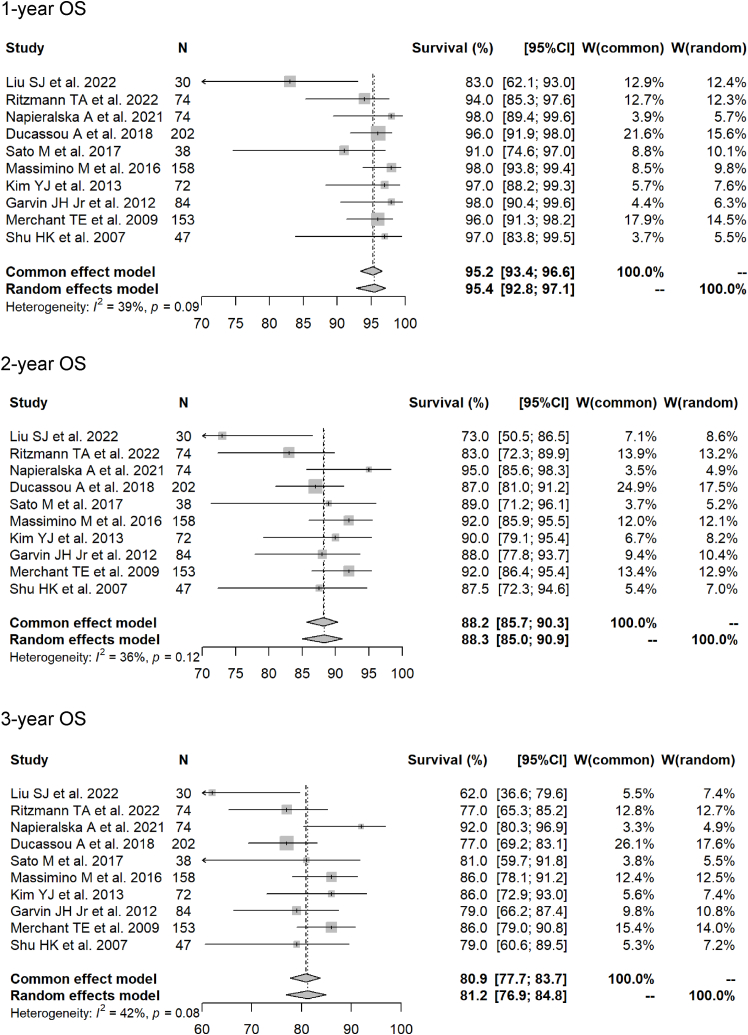

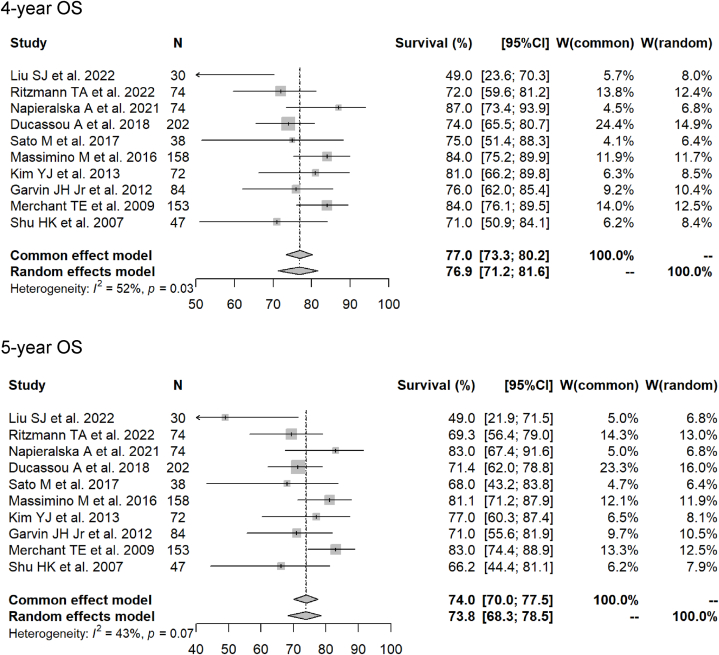
Forest plots of 1- to 5-year overall survival rate for photon radiotherapy.

Using the 15 chosen articles, the meta-analysis showed 1- to 5-year OS rates (photon RT vs. PBT) of 95.4 % (95 % CI 92.8–97.1 %) vs. 97.2 % (95.7–98.2 %) (p = 0.1477); 88.3 % (85.0–90.9 %) vs. 93.5 % (91.4–95.1 %) (p = 0.0026); 81.2 % (76.9–84.8 %) vs. 91.1 % (88.4–93.2 %) (p = 0.0001); 76.9 % (71.2–81.6 %) vs. 86.1 % (81.9–89.4 %) (p = 0.0236); and 73.8 % (68.3–78.5 %) vs. 84.7 % (79.9–88.5 %) (p = 0.0109). Forest plots for each modality for the 1- to 5-year OS rates are shown in Fig. 2, Fig. 3.

**Fig. 4:**
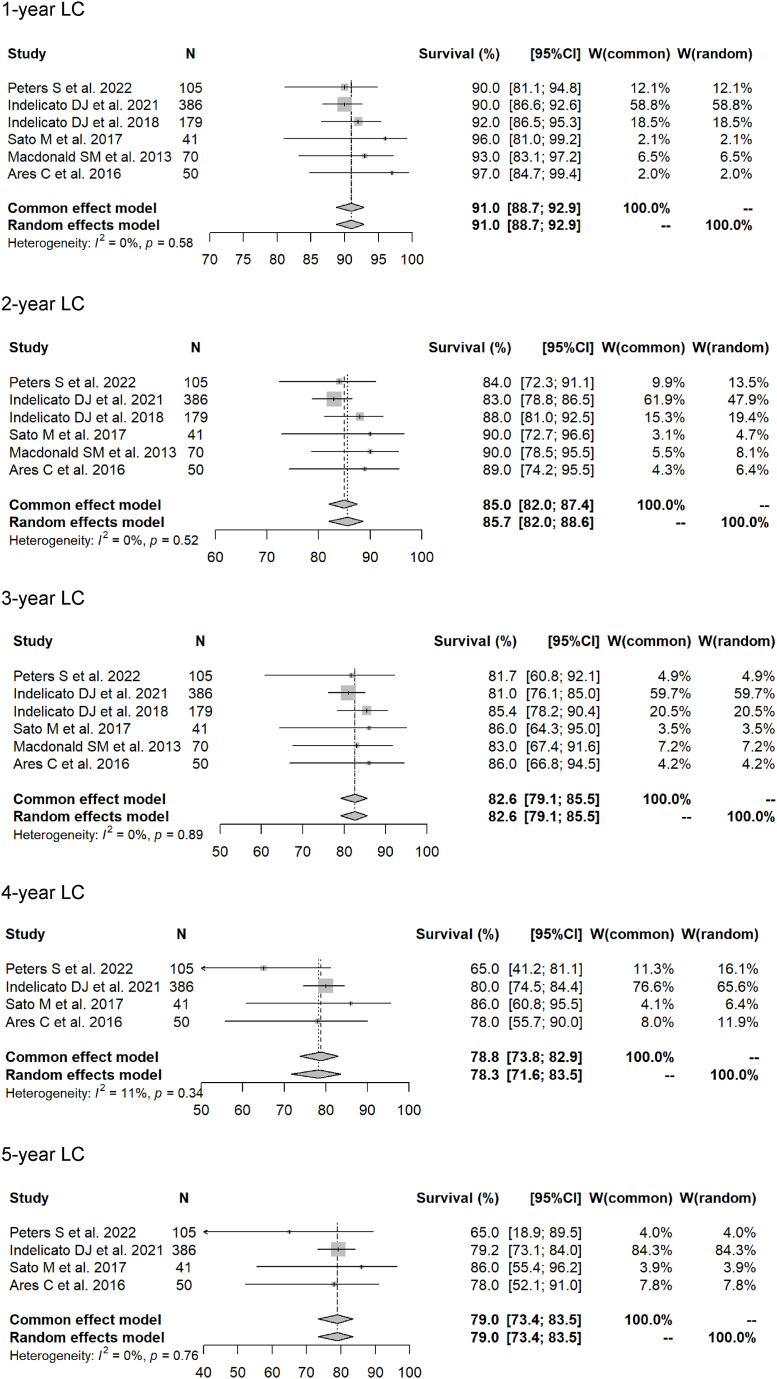
Forest plots of 1- to 5-year local control rate for proton beam therapy.

**Fig. 5:**
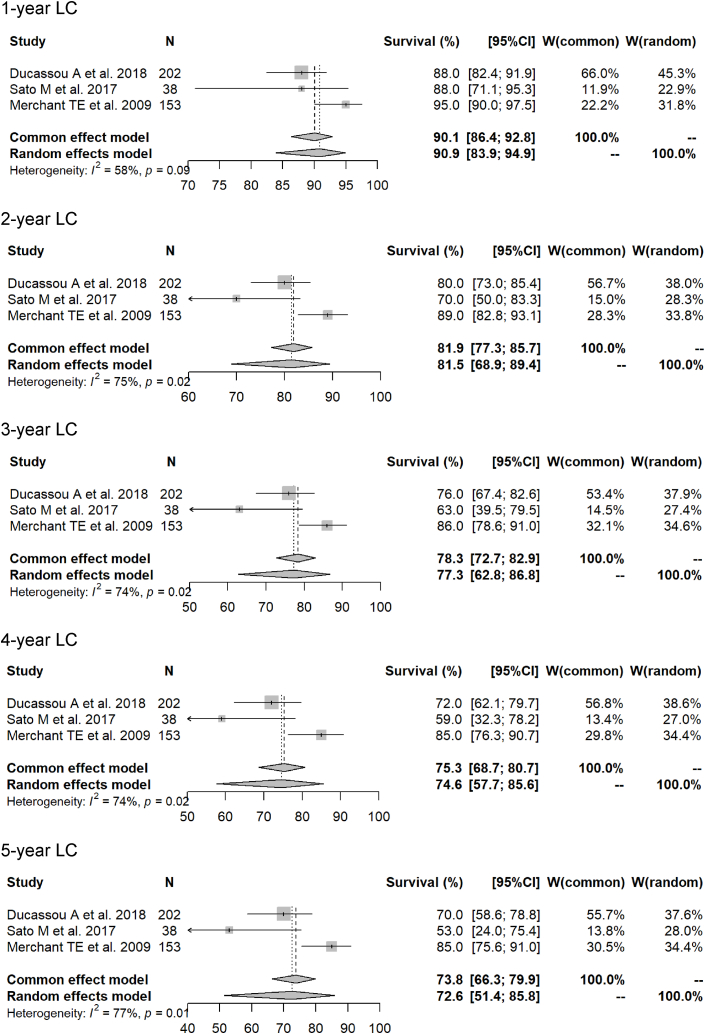
Forest plots of 1- to 5-year local control rate for photon radiotherapy.

The 1- to 5-year LC rates (photon RT vs. PBT) in the meta-analysis were 90.9 % (95 % CI 83.9–94.9 %) vs. 91.0 % (88.7–92.9 %) (p = 0.6691); 81.5 % (68.9–89.4 %) vs. 85.7 % (82.0–88.6 %) (p = 0.2326); 77.3 % (62.8–86.8 %) vs. 82.6 % (79.1–85.5 %) (p = 0.2033); 74.6 % (57.7–85.6 %) vs. 78.3 % (71.6–83.5 %) (p = 0.7189); and 72.6 % (51.4–85.8 %) vs. 79.0 % (73.4–83.5 %) (p = 0.5218). Forest plots for each modality for the 1- to 5-year LC rates are shown in Fig. 4, Fig. 5.

Meta-regression analysis was performed using modality (photon RT vs. PBT), male: female ratio, median age, infratentorial lesion, Grade 3 pathology, concurrent chemotherapy, and complete resection as risk factors, to the extent that information could be obtained from each article. The actual numbers for each factor (photon RT vs. PBT) were: % male 44–70 % (median 56 %) vs. 47–72 % (57 %); age 2.9–8.0 (5.0) vs. 2.6–4.0 (3.5) years; infratentorial lesion rate 58–80 % (64 %) vs. 66–82 % (72 %); Grade 3 21–82 % (49 %) vs. 47–92 % (71 %), concurrent chemotherapy 23–68 % (35 %) vs. 15–86 % (31 %), and complete resection 45–86 % (62 %) vs. 66–93 % (78 %). The total doses of 54–59.4 Gy (median 55.8 Gy) for photon RT and 55.8–59.4 GyE (55.8 GyE) for PBT were almost the same, and therefore, were excluded from analysis. The meta-regression analysis identified relationships of modality (photon RT vs. PBT), age at irradiation, pathology (Grade 2 vs. Grade 3), and tumor removal (complete resection vs. none) with significantly better 3-year OS after PBT and better 1- to 5-year LC at a younger age. The results are shown in Table 2.

**Table 2:** Meta-regressions of potential predictive factors for 1- to 5-year overall survival and 1- to 5-year local control.

Factors	estimate	se	zval	pval	ci.lb	ci.ub
1-year OS						
modality	0.5571	0.3740	1.4895	0.1363	−0.1760	1.2903
age	0.0200	0.1415	0.1417	0.8873	−0.2573	0.2903
Grade3	0.0264	0.0149	1.7709	0.0766	−0.0028	0.0556
GTR	−0.0099	0.0173	−0.5740	0.5660	−0.0438	0.0239
2-year OS						
modality	0.3956	0.2387	1.6571	0.0975	−0.0723	0.8634
age	0.1243	0.0910	1.3666	0.1718	−0.0540	0.3026
Grade3	0.0010	0.0092	0.1127	0.9103	−0.0170	0.0190
GTR	0.0033	0.0106	0.3124	0.7547	−0.0175	0.0241
3-year OS						
modality	0.7013	0.2288	3.0656	0.0022	0.2529	1.1496
age	0.0846	0.0816	1.0363	0.3001	−0.0754	0.2446
Grade3	0.0037	0.0077	0.4745	0.6351	−0.0115	0.0188
GTR	0.0023	0.0094	0.2404	0.8100	−0.0162	0.0207
4-year OS						
modality	0.3902	0.2655	1.4698	0.1416	−0.1301	0.9105
age	0.0749	0.0841	0.8911	0.3729	−0.0899	0.2397
Grade3	0.0014	0.0079	0.1807	0.8566	−0.0141	0.0170
GTR	0.0002	0.0099	0.0252	0.9799	−0.0192	0.0197
5-year OS						
modality	0.4097	0.2570	1.5943	0.1109	−0.0940	0.9134
age	0.0800	0.0822	0.9733	0.3304	−0.0811	0.2412
Grade3	0.0012	0.0077	0.1505	0.8803	−0.0140	0.0163
GTR	−0.0008	0.0099	−0.0776	0.9382	−0.0201	0.0186
1-year LC						
modality	−0.2710	0.2820	−0.9610	0.3365	−0.8237	0.2817
age	0.3600	0.1821	1.9765	0.0481	0.0030	0.7169
Grade3	−0.0025	0.0143	−0.1718	0.8636	−0.0305	0.0256
GTR	−0.0027	0.0191	−0.1392	0.8893	−0.0400	0.0347
2-year LC						
modality	−0.0728	0.2089	−0.3485	0.7274	−0.4823	0.3367
age	0.3173	0.1319	2.4058	0.0161	0.0588	0.5758
Grade3	0.0058	0.0109	0.5310	0.5954	−0.0155	0.0270
GTR	−0.0036	0.0155	−0.2335	0.8153	−0.0341	0.0268
3-year LC						
modality	−0.0220	0.2051	−0.1073	0.9146	−0.4240	0.3800
age	0.3408	0.1317	2.5874	0.0097	0.0827	0.5990
Grade3	0.0013	0.0108	0.1225	0.9025	−0.0198	0.0224
GTR	−0.0124	0.0151	−0.8244	0.4097	−0.0420	0.0171
4-year LC						
modality	−0.2300	0.2972	−0.7740	0.4389	−0.8124	0.3524
age	0.4562	0.1957	2.3312	0.0197	0.0726	0.8397
Grade3	−0.0145	0.0223	−0.6513	0.5148	−0.0583	0.0292
GTR	−0.0642	0.0357	−1.7977	0.0722	−0.1341	0.0058
5-year LC						
modality	−0.1990	0.3300	−0.6031	0.5464	−0.8457	0.4477
age	0.5060	0.2171	2.3307	0.0198	0.0805	0.9315
Grade3	−0.0127	0.0257	−0.4959	0.6200	−0.0631	0.0376
GTR	−0.0576	0.0429	−1.3411	0.1799	−0.1417	0.0266

OS: overall survival, LC: local control.

## 4 Discussion

In this meta-analysis, 1- to 5-year OS and LC rates were compared for PBT and photon RT as postoperative local radiation therapy for pediatric intracranial ependymomas. Forest plots showed that 1- to 5-year OS was better for PBT, whereas 1- to 5-year LC rates were similar for the two modalities. In terms of patient background, Grade 3 cases were more commonly treated with PBT (71 % vs. 49 %) and the complete resection rate tended to be lower in cases treated with photon RT (62 % vs. 78 %). Meta-regression analysis using these factors showed no difference in survival rates by treatment modality, and only 3-year OS rates were better for PBT. The LC rate tended to be better for patients who were younger at the time of irradiation. These results show that PBT gives comparable results to conventional photon RT in postoperative local treatment of intracranial ependymomas in children.

Merchant et al. used CTV and PTV margins of 10 and 3–5 mm, respectively in RT for ependymomas, and similar settings are selected in many local irradiation protocols for both photon RT and PBT [8]. Many of the reports examined in this study used similar CTV (5–10 mm) and PTV (3–5 mm) margins. These studies indicated that postoperative irradiation of 54–59.4 Gy with a margin of about 1 cm is likely to provide equally good LC and OS with photon RT and PBT. However, a drawback of the study was the lack of evaluation of long-term advantages and disadvantages of PBT [28]. Possible benefits of PBT for pediatric tumors include a lower incidence of second cancers and a lower risk of late adverse events such as hearing impairment and reduced pituitary gland function [29,30]. Benefits of PBT for reducing second cancers have been shown in pediatric tumors other than ependymomas, but there is a need for long-term follow-up for accurate assessment of late adverse events [[31], [32], [33]].

Ependymomas are often infratentorial tumors, and risks such as brainstem necrosis are of concern, even with local irradiation [34]. It is generally considered that local irradiation of about 50–60 GyE results in less than 5 % brainstem necrosis. In imaging of the brainstem in 37 cases of PBT and 35 of IMRT for ependymoma, Gunther et al. found that imaging changes were significantly more common with PBT, but that the number of symptomatic cases was similar [35]. In 313 PBT cases (including 73 ependymomas) with the brainstem included in the irradiation range of ≥50.4 GyE, Indelicato et al. found a 2-year late adverse event rate of 3.8 % and a rate of 2.1 % for late adverse events of Grade 3 or higher in the brainstem [36]. Taken together, these reports and the present results suggest that PBT at the same dose as photon RT has almost the same outcomes as photon RT for pediatric intracranial ependymomas, and that there are no PBT-specific late adverse events. There is a need to examine possible reduction of adverse events and the social and economic benefits of use of PBT for intracranial ependymoma. We utilize PBT for almost all pediatric tumors. The Tsukuba Review Project is currently conducting a systematic review and meta-analysis to assess whether PBT and photon RT yield comparable outcomes for other representative pediatric tumors. The results of these analyses will confirm the reproducibility of PBT for pediatric cases. Additionally, long-term adverse events such as secondary cancers are being evaluated in an ongoing prospective registry study in Japan.

## 5 Conclusion

In postoperative local irradiation of ependymomas in children, proton beam therapy had outcomes comparable to those of photon radiotherapy. Further accumulation of cases and follow-up are needed to evaluate the advantages and disadvantages of PBT in terms of adverse events.

## CRediT authorship contribution statement

**Masashi Mizumoto:** Writing – original draft, Formal analysis, Data curation, Conceptualization. **Sho Hosaka:** Data curation, Conceptualization. **Kei Nakai:** Data curation, Conceptualization. **Yinuo Li:** Data curation. **Yoshiko Oshiro:** Data curation. **Takashi Iizumi:** Data curation. **Takashi Saito:** Data curation. **Masako Inaba:** Data curation. **Hiroko Fukushima:** Data curation. **Ryoko Suzuki:** Data curation. **Shosei Shimizu:** Data curation. **Kazushi Maruo:** Formal analysis. **Hideyuki Sakurai:** Writing – review & editing.

## Ethical approval

Not required.

## Data availability statement

All data generated or analyzed during this study are included in this article. Further enquiries can be directed to the corresponding author.

## Funding sources

This work was supported by institutional funds only.

## Declaration of Competing Interest

The authors declare that they have no known competing financial interests or personal relationships that could have appeared to influence the work reported in this paper.
